# Internalization-related host factors of common respiratory viruses

**DOI:** 10.3389/fimmu.2025.1724561

**Published:** 2026-01-16

**Authors:** Qiuchi Lv, Zhengde Xie, Lili Xu

**Affiliations:** 1 Beijing Key Laboratory of Core Technologies for the Prevention and Treatment of Emerging Infectious Diseases in Children, National Clinical Research Center for Respiratory Diseases, National Key Discipline of Pediatrics (Capital Medical University), Beijing Research Center for Respiratory Infectious Diseases, Beijing Pediatric Research Institute, Beijing Children’s Hospital, Capital Medical University, National Center for Children’s Health, Beijing, China; 2 National Key Discipline of Pediatrics (Capital Medical University), Beijing Research Center for Respiratory Infectious Diseases, Beijing Pediatric Research Institute, Beijing, China; 3 Beijing Children’s Hospital, Capital Medical University, National Center for Children’s Health, Beijing, China; 4 Research Unit of Critical Infection in Children, Chinese Academy of Medical Sciences, Beijing, China

**Keywords:** host factors, human adenovirus, respiratory syncytial virus, respiratory virus, virus internalization

## Abstract

Respiratory viruses impose a substantial health burden worldwide, with viral internalization into host cells being the initial step for infection establishment. This process is tightly regulated by the host cellular machinery through two major pathways: receptor-mediated endocytosis and direct membrane fusion. To clarify the role of host factors in these steps, we present human adenovirus and respiratory syncytial virus as representative non-enveloped and enveloped viruses, respectively, as models to elucidate their life cycles, focusing on how host factors mediate their distinct internalization processes. We further categorized the host factors involved in the internalization of other common respiratory viruses, including coronaviruses, influenza A virus, and human metapneumovirus. By analyzing the virus–host interaction mechanisms underlying these processes, this review provides critical insights for developing broad-spectrum antiviral therapies targeting conserved host factors that govern viral internalization.

## 1 Introduction

Respiratory viral infections represent a leading cause of global morbidity and mortality, imposing a substantial burden on public health worldwide. The establishment of respiratory viral infections begins with the critical step of viral internalization, a process following attachment that allows the virus to enter the host cell. By hijacking the host cellular machinery, internalization enables subsequent viral replication and assembly, making it a pivotal phase in the viral life cycle. This entry process is highly dependent on host factors, which are primarily mediated through endocytosis or membrane fusion and additional pathways such as macropinocytosis and clathrin-independent endocytosis. Non-enveloped viruses predominantly utilize endocytosis for host cell entry. In contrast, enveloped viruses enter cells either through direct fusion at the plasma membrane or via endocytosis followed by fusion within endosomal compartments. This fundamental difference in entry mechanisms necessitates the engagement of distinct sets of host factors. As such, host factors represent critical regulatory nodes that extensively govern viral infection and pathogenesis. However, despite their significance, the specific mechanisms and functions of host factors in the internalization of respiratory viruses remain incompletely elucidated, necessitating further systematic investigations. Understanding these host–virus interactions are crucial for the development of novel therapeutic interventions. In this review, we summarize the current knowledge on host factors that are involved in the internalization of common respiratory viruses. We employ human adenovirus (HAdV) and respiratory syncytial virus (RSV) as representative models for non-enveloped and enveloped viruses, respectively, to delineate their life cycles and highlight both the conserved and distinct strategies used by different viral families. The major respiratory virus families discussed in this review, along with their key structural and entry characteristics, are summarized in **Table 1**. Our aim is to provide new perspectives for the development of broad-spectrum antiviral strategies, specifically by targeting conserved host factors that govern viral entry.

**Table 1:** Overview of major respiratory virus families and their structural characteristics.

Virus family	Pathogen	Virion structure	Genome characteristics	Main receptors	Reference
Adenoviridae	HAdV	Non-enveloped with an icosahedral symmetric structure	Linear double-stranded DNA	CAR, DSG2, CD46	(19)
Paramyxoviridae	RSV	Enveloped, spherical or pleomorphic particles	Non-segmented negative-sense RNA	CX3CR1, GAGs	(62)
Coronaviridae	HCoV-229E, HCoV-OC43, MERS-CoV, SARS-CoV, and SARS-CoV-2	Enveloped, spherical or pleomorphic particles	Linear positive-sense RNA	ACE2, APN, DPP4, sialic acid	(75, 129)
Orthomyxoviridae	IAV	Enveloped, spherical or filamentous particles	Segmented negative-sense RNA	Sialic acid	(129)
Paramyxoviridae	HMPV	Enveloped, spherical or pleomorphic particles	Non-segmented negative-sense RNA	HSPGs, DC-SIGN/L-SIGN, Integrins	(130)

## 2 Life cycles of common respiratory viruses

Based on the presence or absence of an outer lipid membrane, viruses are classified as either enveloped or non-enveloped. Once virus enters host cells, the complete process encompassing genome replication and the generation of progeny viruses is termed the replication cycle. Internalization refers to the process by which viruses, following attachment to the host cell membrane, enter the cell through mechanisms such as endocytosis or membrane fusion. Endocytosis refers to the process in which, following virus–cell binding, the plasma membrane invaginates inward to form vesicles that encapsulate viral particles for cellular entry. Notably, non-enveloped viruses predominantly utilize endocytosis to enter host cells. Membrane fusion refers to the process whereby the viral envelope closely engages with the cell membrane and, under the action of fusion proteins, merges with it to release the viral nucleocapsid into the cytoplasm (1). However, enveloped viruses can enter with the direct fusion at the cell-membrane site or after endocytosis and fusion within the endosome in the cytoplasm, such as influenza viruses from the *Orthomyxoviridae* family (2). These distinct internalization mechanisms rely on different sets of host factors. To better understand the impact of host factors on viral infection, this section presents the life cycles of representative respiratory viruses—using RSV as an example of an enveloped virus and HAdV as an example of a non-enveloped virus.

### 2.1 Enveloped viruses: respiratory syncytial virus

RSV is an enveloped, single-stranded, negative-sense RNA virus (3). As a major pathogenic agent, RSV is a leading cause of lower respiratory tract infections such as bronchiolitis and pneumonia in infants and young children (4). RSV is an enveloped, single-stranded, negative-sense RNA virus belonging to the family *Pneumoviridae* and is primarily classified into two subtypes—A and B (3). RSV infection of host cells is mediated mainly by G protein binding to host surface molecules to facilitate viral attachment, while the F protein plays an auxiliary role in the adsorption of viral particles (5). And its infection begins in the nasal epithelial cells of the upper respiratory tract. Following successful infection, the virus can spread from cell to cell, leading to respiratory symptoms (6).

#### 2.1.1 Viral attachment and entry

RSV entry into host cells, the first step of viral entry, is mediated primarily by glycoproteins on the viral surface. The G protein, which is the receptor protein, is responsible for the initial binding of the virion to the host cell surface (5). Through its positively charged domains, the G protein mediates interactions with cell surface glycosaminoglycans (GAGs), including heparan sulfate (7). Most importantly, C-X3-C motif chemokine receptor 1 (CX3CR1) serves as the key receptor in natural infections, mediating RSV attachment through specific binding with the G protein (8). Following attachment, the F protein mediates fusion between the viral envelope and the host cell membrane. Subsequently, the viral ribonucleoprotein complex (RNP)—containing the genomic RNA along with the N, P, and L proteins—is released into the cytoplasm (9). RSV may utilize multiple entry mechanisms. In addition to direct fusion with the plasma membrane, some studies suggest that the virus may also enter cells through endocytic pathways such as macropinocytosis (10, 11).

#### 2.1.2 Viral transcription and replication

Viral RNA replication and transcription in the cytoplasm depend entirely on RNPs. After entering the cytoplasm, the viral RNP uses the genomic RNA (-ssRNA) as a template to initiate transcription (6, 12). Transcription is initiated at the 3′ end of the genome and occurs sequentially. The transcription of each gene is regulated by gene start (GS) and gene end (GE) signal sequences. The polymerase generates 10 capped and polyadenylated subgenomic mRNAs, which are subsequently translated into viral proteins by host ribosomes (13). The replication process involves the synthesis of full-length antigenomic RNA (+ssRNA) as an intermediate, followed by the production of new negative-sense genomic RNA (-ssRNA) from this template. These components are then assembled into nascent viral particles within the cytoplasm (14).

#### 2.1.3 Viral assembly and budding

**Figure 1:**
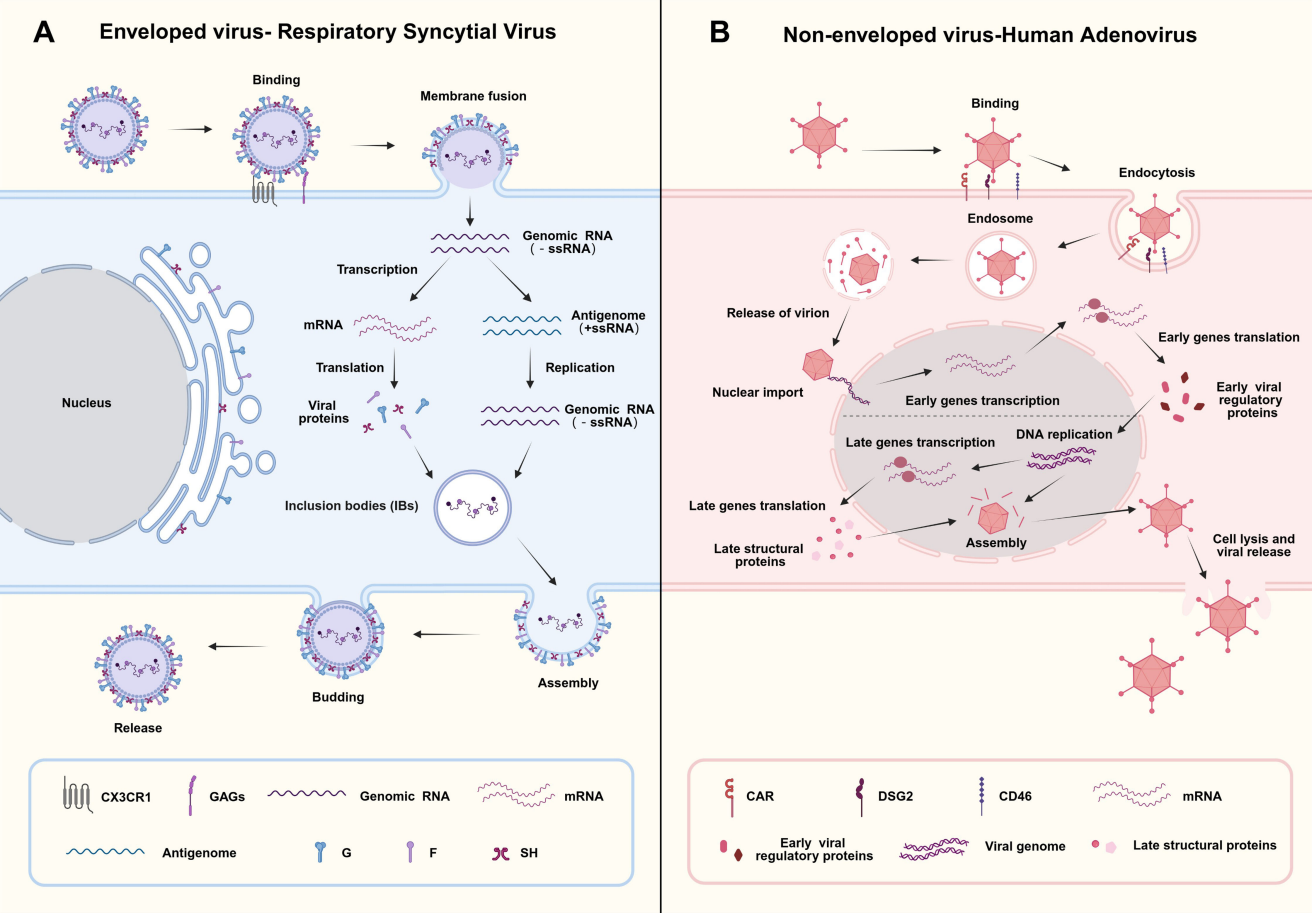
Comparative lifecycle of an enveloped virus (RSV) and a non-enveloped virus (HAdV). Panel **(A)** illustrates the RSV lifecycle: entry via membrane fusion, followed by transcription, translation, inclusion body formation, assembly, and release. Panel **(B)** depicts the HAdV lifecycle: entry via endocytosis, nuclear import of genetic material, gene transcription, translation, virion assembly, and release via cell lysis. Legends detail key molecular components and processes. Created in https://BioRender.com. Agreement number: TS2924UGAV.

The key orchestrator of viral assembly is the M protein. It accumulates in cytoplasmic inclusion bodies (IBs) and interacts with newly formed RNPs to promote virion assembly (15, 16). After the glycoprotein complexes and RNPs are fully assembled, mature viral particles detach from the apical membrane and exit the host cell by budding. Unlike many conventional enveloped RNA viruses, RSV budding does not rely on the host endosomal sorting complexes required for transport (ESCRT) machinery but rather depends on the host apical recycling endosome (ARE) system (17). The M protein of RSV has been confirmed to directly interact with both Rab11a and cytoskeletal components. This interaction likely facilitates the transport of viral components to the apical membrane and promotes the release of viral particles through budding (18). Ultimately, the mature viral particles are released through budding from the host cell membrane and proceed to infect adjacent cells (**Figure 1A**).

### 2.2 Non-enveloped viruses: human adenovirus

HAdV, belonging to the genus *Mastadenovirus* in the family *Adenoviridae*, is a non-enveloped double-stranded DNA virus. It can be classified into seven subgroups (A–G) on the basis of genotype and has 116 serotypes (19, 20). The knob domain of the major capsid fiber protein specifically recognizes receptors on the host cell surface. Different serotypes of HAdV bind to distinct receptors through their fiber proteins, which determines their tissue tropism (21, 22). The life cycle of HAdV is a highly coordinated process that can be broadly divided into three main phases: the entry phase, the early phase, and the late phase (**Figure 1B**).

#### 2.2.1 Entry phase

The virus initiates infection through binding of its fiber protein to receptors on the cell surface. The specific receptor utilized depends on the HAdV genotype. While most HAdV types can utilize coxsackievirus and the adenovirus receptor (CAR), Group B primarily employs desmoglein-2 (DSG2) as its cellular receptor for infection (23). Other utilizable receptors also include integrins, sialic acid, and CD46 (24–26). Following attachment to the cell surface, the virus mediates its internalization through endocytic pathways via interactions between the RGD motif on the penton base and cellular integrins (such as αvβ3 and αvβ5) (27, 28). Different HAdV subtypes utilize distinct pathways for endocytic entry into host cells, including clathrin-mediated endocytosis, caveolin-mediated endocytosis, and macropinocytosis. Specifically, HAdV-C2 and HAdV-C5 employ clathrin-mediated endocytosis (CME) for cellular internalization (29). Reports of HAdV entering host cells via caveolin-mediated endocytosis are relatively limited. Specifically, HAdV-C5 can utilize this pathway to infect U266 cells, whereas HAdV-D37 can enter human corneal fibroblasts through a caveolin-1 (Cav-1)-dependent lipid raft mechanism (30, 31). On the other hand, HAdV-B3 and HAdV-B35 enter cells via macropinocytosis. Through binding to CD46 and integrins, these viruses trigger the downstream activation of Ras-related C3 botulinum toxin substrate 1 (Rac1), p21-activated kinase 1 (PAK1), and C-terminal binding protein 1 (CtBP1), thereby inducing macropinocytosis (32, 33). Within endosomes, the viral capsid undergoes partial disassembly, leading to the exposure of pVI proteins that disrupt the endosomal membrane, thereby releasing viral particles into the cytoplasm. These particles are subsequently transported along microtubules to the nuclear pore complex (NPC), where the viral genome—guided by the core protein pVII containing nuclear localization signals (NLSs)—is ultimately imported into the nucleus (21, 22, 34).

#### 2.2.2 Early phase

Following nuclear entry, viral DNA promptly initiates early gene expression. Nuclear transcription generates viral mRNAs that are then exported to the cytoplasmic translation machinery (19). As a key regulator of early gene transcription, the E1A protein drives aberrant cell cycle activation (35). HAdV E1A bypasses this regulation by sequestering pRb, which inappropriately releases E2F to induce cell cycle gene expression, thereby forcing terminally differentiated cells originally in a quiescent state to re-enter the cell cycle (36, 37). Subsequently, other early genes begin to be expressed. The two proteins encoded by E1B—E1B-55K and E1B-19K—also function to prevent apoptosis in infected cells (38, 39). E2A encodes a single-stranded DNA binding protein (DBP), and E2B encodes a terminal protein (pTP) and viral DNA polymerase, both of which play vital roles in viral genome replication (40–42). The E3 gene encodes multiple immunomodulatory proteins that help the virus evade host immune recognition and contribute to the establishment of persistent HAdV infection (43). The E4 gene encodes multiple protein products that regulate transcription, cell cycle progression, cell signaling, and DNA repair while also promoting the transition from the early to late phase of infection (44, 45). During the early phase, translated early viral regulatory proteins are transported back to the nucleus to participate in the expression of late genes.

#### 2.2.3 Late phase

The late phase is driven by the major late promoter (MLP), which generates a large number of mRNAs that encode viral structural proteins through alternative splicing (46). Within the nucleus, structural proteins are progressively assembled into procapsids, while the viral genome is packaged with the assistance of the core proteins pVII and pV (47). The adenovirus protease (AVP) that is encoded by the L3 gene is essential for the production of infectious viral particles, as it proteolytically processes multiple viral proteins (48). During late-stage infection, massive production of progeny viruses occurs. The adenovirus death protein (ADP) encoded by the E3 region mediates nuclear envelope destabilization and cellular lysis, thereby facilitating the release of viral particles (49, 50).

## 3 Internalization-related host factors of common respiratory viruses

Viral internalization, a vital step in the life cycle of common respiratory viruses, is highly dependent on host factors that mediate pathways such as endocytosis and membrane fusion. Distinct sets of host factors are engaged by non-enveloped and enveloped respiratory viruses to facilitate their entry. We have compiled a summary of host factors associated with the internalization of common respiratory viruses and provided a concise description of their mechanisms, elucidating these host-virus interactions lays the foundation for developing novel broad-spectrum antiviral strategies (**Table 2**).

**Table 2:** Host factors for the internalization of common respiratory viruses.

Virus	Function (promote/inhibit infection)	Host factors	Mechanism	Reference
RSV	Promote	ATP1A1	Activated by RSV; transmits signals via phosphorylated Src kinase to activate EGFR, inducing cytoskeletal rearrangement for macropinocytosis	(61, 62)
		Rab5	RSV enters via micropinocytosis, depending on Rab5-positive early macropinosomes for F protein cleavage (enabling infectivity)	(10)
	Inhibit	IFITMs	Alters cell membrane properties (fluidity, curvature) to disrupt viral membrane fusion; suppresses entry and early replication	(56, 57)
HAdV	Promote	IL-8	Enhances localization of HAdV receptors (CAR, integrins) at apical membrane of polarized epithelial cells to boost viral entry	(64–67)
		ALCAM	Interacts with fiber protein of HAdV-B7; promotes internalization via ALCAM-DSG-2-EndoA3 axis	(62)
CoVs	Promote	Fruin	Cleaves SARS-CoV-2 spike protein at S1/S2 junction; induces conformational changes to expose receptor-binding domain, enhancing membrane fusion	(88, 89)
		TMPRSS2	Cleaves ACE2 (promoting viral uptake) and spike protein (exposes fusion peptide); facilitates plasma membrane fusion to bypass NCOA7 restriction	(74, 88, 90, 91)
		CTSL	Cleaves spike protein at S2′ site in endosomes (requires acidification); exposes fusion peptide to enable membrane fusion	(74, 92)
		Polyamines	Synthesized via ODC1; facilitate viral binding and entry (inhibited by DFMO)	(97)
	Inhibit	GPI and LY6E	Regulates downstream LY6E to disrupt spike protein-mediated membrane fusion at endosomal/plasma membranes Modulates cell membrane properties/immune responses to block spike-mediated membrane fusion	(77–80)
		CH25H	Catalyzes 25HC production; 25HC activates ACAT to deplete plasma membrane cholesterol, blocking viral membrane fusion	(84)
		SERINC5	Incorporated into SARS-CoV-2 progeny virions; binds spike protein to impede membrane fusion (inhibition countered by ORF7a)	(86)
		PSGL-1	Structurally hinders interaction between virions and target cells, inhibiting binding/entry of spike-bearing VLPs	(87)
		GILT	Reduces disulfide bonds in viral envelope glycoproteins; disrupts membrane fusion or causes glycoprotein degradation	(95)
IAV	Promote	SLC35B4 and AGRN	Mediates internalization of diverse IAV subtypes; prevents excessive accumulation of nonheparan sulfate-modified AGRN (avoids AP2B1 degradation) Interacts with HA1 of IAV HA protein; recruits AP2B1 to initiate viral internalization	(100)
		Paxillin	Positively regulates IAV replication; influences viral internalization and/or endosomal trafficking (no effect on membrane fusion)	(102)
		Epsin1 and Neo1	Mediates interaction between its UIMs and ubiquitinated viral receptors; promotes viral binding to clathrin and CCP formation for entry Interacts with IAV via N-linked glycosylations; colocalizes with incoming virus early postinfection to facilitate entry	(107, 109)
		EGFR	Activated by IAV attachment; triggers lipid raft aggregation and EGFR autophosphorylation; coordinates with clathrin/Cav-1 to promote internalization	(112, 113)
		FFAR2	Activated by IAV HA/M2; phosphorylated by GRKs to recruit β-arrestin1; β-arrestin1 binds AP2B1 to promote clathrin-coated pit maturation for endocytosis	(115)
		mGluR2	IAV HA interacts with mGluR2; mGluR2 activates KCa1.1 channel, promoting F-actin polymerization to assist IAV’s clathrin-mediated endocytosis	(116)
	Inhibit	IFITMs	Inhibits the membrane fusion of IAV through its amphipathic helix	(103)
		SERINC5	Suppresses IAV HA protein-mediated membrane fusion and its restrictive effect is modulated by HA glycosylation	(104)
		RABGAP1L	Disrupts IAV membrane fusion and blocks infection by impairing endosomal maturation and trafficking, thereby interfering with viral colocalization with early endosomes	(106)
HMPV	Promote	TMPRSS2	Cleaves HMPV F protein; promotes membrane fusion and viral multiplication	(120)
	Inhibit	IFITM3	Blocks HMPV F protein-mediated membrane fusion; Y20A mutation alters its localization to enhance restriction	(119)

### 3.1 Respiratory syncytial virus

#### 3.1.1 Interferon-inducible transmembrane proteins

Interferon-inducible transmembrane proteins (IFITMs) are a class of host restriction factors that are induced by interferon stimulation and include IFITM1, IFITM2, IFITM3, IFITM5, and IFITM10. They play a critical role in innate immunity and have been demonstrated to restrict the replication of diverse viruses, such as influenza A virus (IAV), dengue virus (DENV), west Nile virus (WNV), and SARS-CoV-2. Additionally, IFITMs are involved in crucial biological processes, including cell signaling, adhesion, tumorigenesis, and immune regulation (51–53). IFITMs have been suggested to disrupt viral membrane fusion by altering cellular membrane properties such as fluidity and curvature (54, 55). Research has indicated that IFITMs primarily suppress RSV infection by interfering with the viral entry and early replication stages but have no inhibitory effect on the viral attachment phase (56). IFITM1 localized to the plasma membrane directly blocks viral entry into host cells by interfering with the membrane fusion process of RSV. It contains a conserved cytoplasmic intracellular loop (CIL), which serves as the core structure for the plasma membrane localization of IFITM1. CIL domain mutations can disrupt the protein’s ability to anchor to the plasma membrane, resulting in the loss of its antiviral activity (57).

#### 3.1.2 ATP1A1

ATP1A1, which consists of 10 transmembrane domains, forms the core of the Na^+^, K^+^-ATPase complex. It is responsible for maintaining the Na^+^/K^+^ gradient across the plasma membrane and plays a vital role in ion transport, electrolyte balance, and fluid homeostasis. ATP1A1 interacts with the cellular kinase c-Src via its cytoplasmic tail. Activated c-Src can mediate the phosphorylation and activation of epidermal growth factor receptor (EGFR) in an EGF-independent manner, thereby inducing macropinocytosis (58–60). In the early stages of RSV infection, the virus triggers the activation and aggregation of ATP1A1 within the plasma membrane. ATP1A1 subsequently transmits signals through phosphorylated Src kinase, leading to EGFR activation via phosphorylation at Tyr845. The activated EGFR signaling cascade induces cytoskeletal rearrangement, which promotes macropinocytosis and facilitates the internalization of RSV into host cells (61, 62).

#### 3.1.3 Rab5

Rab5 belongs to the Rab subfamily of small GTPases that are involved in various cellular processes, such as cell growth, differentiation, intracellular transportation, and signal transduction (63). Studies have indicated that RSV can enter host cells through the macropinocytosis pathway and its infection depends on the environment of Rab5-positive early macropinosomes. The F protein of RSV is critical for membrane fusion and infection, undergoing two cleavages. The second cleavage occurs after the virus enters host cells via the macropinocytosis pathway: within Rab5-positive early macropinosomes, the soluble 27 amino acid peptide (p27) of the F protein is removed, exposing the fusion peptide at its N-terminus. This activates the F protein’s membrane fusion ability, mediating the fusion between the viral envelope and the macropinosome membrane to release the viral genome into the cytoplasm. Rab5 localizes to early macropinosomes, providing a suitable microenvironment for the second cleavage (10).

### 3.2 Human adenovirus

#### 3.2.1 Interleukin-8

Interleukin-8 (IL-8), also known as CXCL8, is a crucial inflammatory cytokine that belongs to the chemokine family. Studies have demonstrated that as a key component of the innate immune system, IL-8 can increase the localization of HAdV-C5 receptors (CAR and integrins) at the apical membrane of polarized epithelial cells. This mechanism promotes viral entry into host cells and consequently increases cellular susceptibility to viral infection (64, 65). Additionally, the early protein E1A of HAdV can stimulate host cells to secrete IL-8, establishing a positive feedback loop that enables the virus to evade the innate immune response of the host cells (66, 67).

#### 3.2.2 Activated leukocyte cell adhesion molecule

The activated leukocyte cell adhesion molecule (ALCAM), also known as CD166, is a transmembrane immunoglobulin-like protein that is present on the cell membrane surface and belongs to the immunoglobulin superfamily (IgSF). It plays significant roles in cell–cell interactions, cell migration, and immune regulation and is also associated with nervous system development (68–70). Furthermore, ALCAM is highly expressed in various cancer tissues, and its expression levels may be associated with prognostic outcomes (71).

ALCAM has been identified as an entry factor for HAdV-B group viruses that are associated with severe community-acquired pneumonia (SCAP). It can interact with the fiber protein of HAdV-B7 and promote the internalization of HAdV-B7 through the ALCAM-DSG-2-EndoA3 axis without affecting viral attachment. Notably, infection by HAdV-B35 is not reduced in ALCAM-knockout cells, suggesting that ALCAM may facilitate viral internalization through alternative mechanisms (72).

### 3.3 Coronaviruses

Coronaviruses (CoVs) are enveloped, positive-sense single-stranded RNA viruses with broad host tropism. The subfamily *Orthocoronavirinae* within the family *Coronaviridae* consists of four genera: α-, β-, γ-, and δ-coronaviruses. Several endemic human α- and β-coronaviruses—including α-coronaviruses HCoV-229E and HCoV-NL63, as well as β-coronavirus HCoV-OC43—typically induce mild common colds, whereas the emerging β-coronaviruses (SARS-CoV, MERS-CoV, and SARS-CoV-2) are capable of causing severe respiratory diseases (73, 74). HCoV-NL63, SARS-CoV, and SARS-CoV-2 primarily utilize angiotensin-converting enzyme 2 (ACE2) as their main receptor; HCoV-229E employs aminopeptidase N (APN) as its primary receptor. MERS-CoV uses dipeptidyl peptidase 4 (DPP4) and HCoV-OC43 predominantly binds to sialic acid (75).

#### 3.3.1 Glycosylphosphatidylinositol and lymphocyte antigen 6 family member E

Glycosylphosphatidylinositol (GPI) is a lipid anchor for many cell-surface proteins (76). It can restrict infections by multiple coronaviruses—including SARS-CoV-2, HCoV-229E, and HCoV-OC43—by restricting viral entry through the disruption of spike protein-mediated membrane fusion at both the endosomal and plasma membranes. The GPI biosynthesis pathway regulates the downstream effector lymphocyte antigen 6 family member E (LY6E) to restrict coronavirus infection (77). LY6E is a GPI-anchored protein that functions as a downstream effector of the GPI pathway. It specifically interferes with membrane fusion by modulating host cell membrane properties and immune responses. This disruption of spike-mediated membrane fusion results in antiviral activity against multiple coronaviruses, including HCoV-229E, HCoV-OC43, MERS-CoV, SARS-CoV, and SARS-CoV-2 (78–80).

#### 3.3.2 Cholesterol 25-hydroxylase

Cholesterol 25-hydroxylase (CH25H), an interferon-inducible gene, is a vital element in cellular cholesterol metabolism (81). CH25H acts as a host restriction factor that suppresses the replication of diverse viruses, including vesicular stomatitis virus (VSV), herpes simplex virus (HSV), human immunodeficiency virus (HIV), Ebola virus, IAV, and Zika virus (ZIKV) (82, 83). CH25H exerts its antiviral effect through hydroxylase activity, which catalyzes the production of 25-hydroxycholesterol (25HC).25HC triggers the depletion of accessible cholesterol from the plasma membrane by activating acyl-CoA:cholesterol acyltransferase (ACAT). This cholesterol depletion in the plasma membrane blocks coronavirus entry and spike protein-mediated membrane fusion. The precise mechanism through which ACAT activation leads to cholesterol removal from the plasma membrane remains incompletely understood. Given the crucial roles of cholesterol in membrane fluidity and polarity, it is suggested that alterations in cholesterol levels may modulate the conformation or distribution of viral receptors or other host membrane proteins, thereby potentially affecting viral entry. This mechanism enables broad-spectrum inhibition of entry for multiple coronaviruses, including MERS-CoV, SARS-CoV, and SARS-CoV-2 (84). Given that both the antiviral activity of CH25H and its catalytic product 25HC are endogenously occurring molecules with favorable safety profiles, they hold promising potential for therapeutic development against viral infections.

#### 3.3.3 Serine incorporator 5

Serine incorporator 5 (SERINC5) is a multispan transmembrane protein that belongs to the serine incorporator family and is thought to play important roles in sphingolipid and phosphatidylserine biogenesis. It is known as a restriction factor for retroviruses, such as HIV-1 and murine leukemia virus (MLV) (85). In a previous study, during assembly in producer cells, SERINC5 is incorporated into budding HIV-1 particles, thereby inhibiting subsequent viral entry into target cells. Similarly, in SARS-CoV-2, SERINC5 can also be incorporated into its progeny virions. By binding to the SARS-CoV-2 spike protein, SERINC5 impedes spike-mediated membrane fusion and consequently blocks viral entry. Notably, this inhibitory effect can be counteracted by the SARS-CoV-2 accessory protein ORF7a (86).

#### 3.3.4 P-selectin glycoprotein ligand-1

P-selectin glycoprotein ligand-1 (PSGL-1) is a cell surface glycoprotein that binds to P-, E-, and L-selectins to mediate the tethering and rolling of immune cells on the surface of the endothelium for cell migration into inflamed tissues. PSGL-1 has been identified as an interferon-γ-regulated factor that restricts HIV-1 infectivity and has recently been shown to possess broad-spectrum antiviral activity. Research has indicated that PSGL-1 inhibits the binding and entry of virus-like particles (VLPs) bearing SARS-CoV and SARS-CoV-2 spike proteins. This inhibition likely occurs because PSGL-1 structurally hinders virion interactions with target cells and blocks the virus from engaging its ACE2 receptor, thereby suppressing viral internalization (87).

#### 3.3.5 Furin

Furin, a member of the proprotein convertase (PC) family, is a calcium-dependent serine protease that is widely distributed in the Golgi apparatus, secretory pathways, and plasma membrane. Studies have revealed that the SARS-CoV-2 spike protein contains a unique polybasic cleavage site at the S1/S2 junction, which enables specific recognition and cleavage by furin. This proteolytic processing induces conformational changes in the S protein, leading to exposure of the receptor-binding domain and enhancement of viral membrane fusion, thereby significantly increasing viral infectivity. Notably, furin can synergize with transmembrane serine protease 2 (TMPRSS2) and cathepsin L to coordinately regulate the cellular entry pathways of SARS-CoV-2 (88, 89).

#### 3.3.6 Transmembrane serine protease 2

TMPRSS2, a type II transmembrane serine protease, cleaves both the SARS-CoV-2 receptor ACE2 and the viral spike protein. On the one hand, its proteolysis of ACE2 promotes viral uptake; on the other hand, cleavage at the S2′ site of the S protein exposes the fusion peptide, thereby triggering fusion between the viral envelope and the host cell membrane (74, 90, 91). Furthermore, TMPRSS2 facilitates SARS-CoV-2 fusion at the plasma membrane, thereby enabling the virus to bypass the restriction mediated by Nuclear Receptor Coactivator 7 (NCOA7) and evade innate immune constraints (88).

#### 3.3.7 Cathepsin L

Cathepsin L (CTSL), a member of the lysosomal cysteine protease family, primarily functions to degrade protein antigens derived from pathogens that are internalized via endocytosis. Notably, CTSL can also trigger proteolysis at the S2′ site of the viral spike protein, leading to the exposure of the fusion peptide and subsequent membrane fusion. This activation of CTSL is associated with the endosomal viral entry pathway and requires endosomal acidification to accomplish membrane fusion (74, 92).

#### 3.3.8 Gamma-interferon-inducible lysosomal thiol reductase

Gamma-interferon-inducible lysosomal thiol reductase (GILT), also named IFI30, is a soluble thiol reductase that is sorted by the mannose 6-phosphate receptor pathway to endocytic compartments and ultimately transported into the lysosome, where it provides an optimal low pH for thiol reductase activity (93). It plays important roles in antigen presentation and tumor immunity and restricts viral infection through multiple mechanisms (94). GILT facilitates the unfolding of internalized proteins containing disulfide bonds in lysosomes, and the reduction in disulfide bonds in the envelope glycoproteins of incoming virions by GILT may result in glycoprotein or interruption of glycoprotein-mediated membrane fusion and consequently inhibit the entry of SARS-CoV, as well as Ebola virus and Lassa fever virus (95).

#### 3.3.9 Polyamines

Polyamines are small aliphatic metabolites that are synthesized by mammalian cells to support cellular processes such as cell cycling, transcription, and translation (96). Polyamines are synthesized under the catalysis of the ornithine decarboxylase 1 (ODC1) and facilitate coronavirus binding and entry. The ODC1 inhibitor difluoromethylornithine (DFMO) significantly inhibits the binding and entry of both HCoV-NL63 and SARS-CoV-2. Polyamines are critical for coronavirus replication and represent highly promising drug targets for addressing current and future coronavirus outbreaks (97).

### 3.4 Influenza A virus

Influenza A virus is an enveloped, negative-sense, segmented single-stranded RNA virus that belongs to the family *Orthomyxoviridae* and has a broad host range. IAVs can be classified into numerous subtypes on the basis of the antigenic properties of two key surface glycoproteins: hemagglutinin (HA) and neuraminidase (NA) (98). As the initial step of the IAV replication cycle, the binding of viral HA to sialic acid receptors on the cell surface triggers receptor-mediated endocytosis of the virus.

#### 3.4.1 Solute carrier family 35 member B4 and agrin

Solute carrier family 35 member B4 (SLC35B4), a key member of solute carrier family 35 (SLC35), belongs to the SLC35B subfamily. It encodes a bifunctional nucleotide sugar transporter that is specifically responsible for transporting uridine diphosphate N-acetylglucosamine (UDP-GlcNAc) and uridine diphosphate xylose (UDP-xylose). Agrin (AGRN) is a secreted extracellular matrix heparan sulfate proteoglycan (99). Research has indicated that SLC35B4 mediates the internalization of diverse IAV subtypes. Moreover, AGRN interacts with the HA1 subunit of the viral HA protein and recruits the adaptor protein 2 subunit beta 1 (AP2B1) to initiate the internalization process. When SLC35B4 is deficient, nonheparan sulfate-modified AGRN accumulates excessively, leading to the degradation of AP2B1 via the ubiquitin–proteasome pathway, thereby suppressing IAV internalization (100).

#### 3.4.2 Paxillin

Paxillin is a multifunctional and multidomain focal adhesion adapter protein that plays important roles in cell motility, adhesion, cancer development and signal transduction (101). Four splice isoforms of paxillin (α, β, γ, and δ) have been reported. Paxillin, which is encoded by the PXN gene, positively regulates IAV replication—a finding that was systematically validated through both *in vitro* cell experiments and *in vivo* mouse models. Moreover, while paxillin does not affect viral membrane fusion or subsequent infection steps, it may influence viral internalization and/or endosomal trafficking. Additionally, paxillin might impact the endosome-dependent entry of other internalized viruses, such as VSV (102).

#### 3.4.3 IFITM3

IFITM3 also functions as a host restriction factor against IAV. It suppresses IAV infection by inhibiting HA-mediated membrane fusion, and an amphipathic helix within IFITM3 is essential for this antiviral activity (103).

#### 3.4.4 Serine incorporator 5

As previously mentioned, SERINC5 also suppresses IAV infection by inhibiting HA-mediated membrane fusion. Moreover, HA glycosylation modulates the sensitivity of IAV to SERINC5 restriction (104). Owing to its broad-spectrum antiviral activity, SERINC5 is recognized as a pivotal target for investigating virus–host interactions and developing novel antiviral strategies.

#### 3.4.5 RAB GTPase activating protein 1 like

RAB GTPase activating protein 1-like (RABGAP1L), also known as TBC1D18 or HHL, belongs to the Tre2/Bub2/Cdc16 domain family of proteins. It primarily regulates membrane-bound small GTPase proteins, termed RAB proteins (2). RABGAP1L plays a critical role in various biological processes, including intracellular trafficking and tumor proliferation (105). RABGAP1L overexpression disrupted normal endosomal function during IAV entry, leading to the prevention of IAV particle fusion with cellular membranes. Furthermore, overexpression of RABGAP1L led to a general reduction in the colocalization of IAV with early endosome antigen 1 (EEA1), a specific marker of early endosomes. In summary, RABGAP1L likely affects viral internalization by regulating endosomal maturation and trafficking (106).

#### 3.4.6 Epsin1 and neogenin

Epsin1 belongs to the epsin protein family and is a multifunctional binding endocytic adaptor. Epsin1 facilitates the internalization of IAV by mediating the interaction between its ubiquitin-interacting motifs (UIMs) and ubiquitinated receptors on the viral surface. This process promotes viral binding to clathrin and the formation of clathrin-coated pits (CCPs), ultimately enabling IAV entry (107). Neogenin (Neo1) is a multifunctional transmembrane receptor that belongs to the immunoglobulin superfamily (108). Research has demonstrated that Neo1 is a potential IAV internalization receptor. Neo1 interacts with IAV through its N-linked glycosylations, colocalizes with the incoming virus early after infection, affects viral entry, and its depletion impairs IAV entry (109).

#### 3.4.7 Epidermal growth factor receptor

EGFR is a transmembrane receptor tyrosine kinase that belongs to the receptor tyrosine kinase (RTK) family and is involved in various stages of viral infection, including viral entry, replication, and immune evasion from the host immune response (110, 111). It can be activated by IAV attachment and is involved in promoting the initial internalization of IAV into host cells. Upon contact and binding of IAV to the host cell membrane, lipid rafts accumulate, leading to the activation of EGFR and the induction of its autophosphorylation. During this process, clathrin and caveolin-1 act as additional regulatory factors that mediate IAV internalization in coordination with EGFR (112, 113).

#### 3.4.8 Free fatty acid receptor 2

Free fatty acid receptor 2 (FFAR2) (also known as GPR43), a member of the free fatty acid receptor (FFAR) family, is classified as a rhodopsin-like receptor. FFAR2 affects numerous physiological functions, including the regulation of energy metabolism, the modulation of inflammatory responses, and gut motility (114). The activation signal of FFAR2 is triggered through interactions with the viral HA and M2 proteins. As a G protein-coupled receptor (GPCR), FFAR2 is phosphorylated by GPCR kinases, leading to the specific recruitment of β-arrestin1. β-Arrestin1 then binds to AP2B1, promoting the maturation of clathrin-coated pits. This process ultimately encloses IAV into endosomes, completing viral internalization. The FFAR2–β-arrestin1–AP2B1 signaling cascade is necessary for the efficient endocytosis of IAV into host cells (115).

#### 3.4.9 Metabotropic glutamate receptor 2

Metabotropic glutamate receptor 2 (mGluR2) is a class C member of the GPCR superfamily and plays a critical role in regulating neurotransmitter signaling in the central nervous system. Recently, mGluR2 has been identified as an endocytic receptor for IAV. IAV directly interacts with mGluR2 via its HA protein and initiates CME, enabling the virus to attach to the cell surface and trigger internalization. Upon binding to IAV, mGluR2 interacts with and activates calcium-activated large-conductance potassium channels (KCa1.1). Activated KCa1.1 induces F-actin polymerization, which facilitates the maturation of clathrin-coated pits, thereby assisting in the completion of the CME process and allowing viral entry into the cell. Multiple subtypes of IAV can utilize mGluR2 as an endocytic receptor, with their CME processes being regulated by KCa1.1 (116).

### 3.5 Human metapneumovirus

HMPV, classified within the *Pneumoviridae* family, is an enveloped virus containing a non-segmented negative-sense RNA genome. HMPV can be divided into two major genotypes, A and B, which are further categorized into six subgenotypes, namely, A1, A2a, A2b, A2c, B1, and B2, on the basis of the sequence variations of the G and F proteins (117). Following attachment and membrane fusion mediated by the G and F glycoproteins, the viral genome is released into the host cell, initiating its transcription and replication. HMPV primarily utilizes heparan sulfate proteoglycans (HSPGs), dendritic cell-specific intercellular adhesion molecule-3-grabbing non-integrin and liver/lymph node-specific ICAM-3 grabbing non-integrin (DC-SIGN/L-SIGN), and integrins as its main receptors (118).

#### 3.5.1 IFITM3

IFITM3 was the first host restriction factor identified for HMPV. Similarly, IFITM3 can block membrane fusion mediated by the hMPV fusion protein. IFITM3 contains a four-amino-acid YxxΦ endocytic signal (^20^-YEML-^23^) at residues 20-23, which mediates its trafficking from the plasma membrane to endosomes and lysosomes. Mutation of IFITM3-Y20A alters the subcellular localization of IFITM3 and significantly enhances its restriction of HMPV (119).

#### 3.5.2 TMPRSS2

In Vero cells constitutively expressing TMPRSS2 (Vero-TMPRSS2) and green fluorescent protein-expressing HMPV, TMPRSS2 efficiently supports the cleavage of the HMPV F protein and HMPV multiplication (120). This kind of cleavage may promote viral membrane fusion, as well as SARS-CoV-2 infection.

## 4 Prospects

Respiratory viruses encompass a wide spectrum of pathogens. With continuous viral circulation and the emergence of novel agents, they remain a major threat to public health. Among the several viruses we have discussed, studies on IAV and SARS-CoV-2 are relatively extensive, with targeted therapeutics and preventive vaccines already on the market (121–123). Monoclonal antibodies for the treatment of RSV and vaccines targeting infants and the elderly have been marketed, but specific therapeutic drugs are not yet available (124). However, for other CoVs, such as HAdV and HMPV, no safe or effective targeted drugs or vaccines are currently available (19, 125). The varying progress in understanding the pathogenic mechanisms of common respiratory viruses has led to disparities in drug and vaccine development, posing a significant obstacle to the development of broad-spectrum antiviral therapies. Despite these challenges, respiratory viruses share common features in their infection of host cells. Viral internalization constitutes the initial step in this process. Blocking this internalization can fundamentally prevent viral infection at its root. Therefore, research on host factors that are related to internalization is highly valuable for the development of broad-spectrum anti-respiratory virus drugs and vaccines.

IFITMs restrict viral entry by altering membrane fluidity and curvature, which are effective for RSV, IAV, and HMPV. TMPRSS2 promotes entry by cleaving viral fusion proteins, which are critical for CoVs and HMPV. They both play pivotal roles in the internalization of respiratory viruses. IFITMs, as naturally occurring antiviral proteins, represent a promising direction for antiviral drug development by upregulating their expression or enhancing their function. Recently, a clinical trial of the use of the IFNα1b inhalation solution GB05 for treating RSV infection has been conducted in China (126). IFN can activate the transcription of IFITMs, which are expected to be broad-spectrum antiviral agents (127). Significant progress has also been made in the development of TMPRSS2 inhibitors, with several promising drug candidates emerging. Conventional TMPRSS2 inhibitors, such as Camostat and Nafamostat, have demonstrated modest efficacy in clinical trials because of their rapid degradation *in vivo*. Recent studies have shown that the novel inhibitor TMP1 simultaneously targets both the viral protease M^pro^ and host protein TMPRSS2, resulting in remarkable anti-coronavirus effects in mouse models (128). Whether TMP1 can be repurposed for other respiratory viruses that also utilize TMPRSS2 as an internal host factor, such as HMPV, remains unclear. **Table 3** summarizes the current potential antiviral studies targeting the aforementioned host factors. A deeper understanding of these targets, along with the development of antiviral analogs and small-molecule inhibitors against pro-infection factors, can offer novel strategies for panrespiratory virus prevention and treatment.

**Table 3:** Antiviral research advances in targeting host factors for respiratory virus internalization.

Host factors	Antiviral strategy	Targeting respiratory viruses	Mechanism	Reference
IFITMs	IFNα1b inhalation solution GB05	RSV	IFNα1b inhalation solution GB05 for treating RSV infection, can activate the transcription of IFITMs	(126, 127)
ATP1A1	Cardiotonic steroid ouabain and the digitoxigenin derivative PST2238 (Rostafuroxin)	RSV	Ouabain and PST2238 bind to the extracellular domain of ATP1A1, thereby inhibiting RSV uptake.	(61)
Furin	furin inhibitors decanoyl-RVKR-chloromethylketone (CMK) and naphthofluorescein	SARS-CoV-2	CMK and naphthofluorescein inhibit TMPRSS2 by blocking its serine protease activity, thereby preventing the cleavage of the SARS-CoV-2 spike protein at the S2’ site and suppressing viral entry into host cells	(131)
TMPRSS2	Conventional TMPRSS2 inhibitors Camostat and Nafamostat, the novel inhibitor TMP1	CoVs	The inhibitors directly inhibit the serine protease activity of TMPRSS2, preventing its cleavage of the S2’ site in the S protein and thereby suppressing the internalization of CoVs	(128)
CTSL	CTSL inhibitors Z-FA-FMK, SM141, SM142 and glycopeptide antibiotic Teicoplanin	SARS-CoV-2	They can suppress CTSL activity by directly targeting its proteolytic domain, thereby blocking CTSL-mediated cleavage of viral spike proteins and inhibiting the endosomal entry of SARS-CoV-2	(132–134)
CH25H	CH25H and its endogenous product 25HC	CoVs	CH25H can catalyzes 25HC production and 25HC activates ACAT to deplete plasma membrane cholesterol, blocking viral membrane fusion	(84)
Polyamines	DFMO	CoVs	DFMO inhibits ODC1, the rate-limiting enzyme in polyamine biosynthesis, thereby depleting intracellular polyamines and suppressing the internalization of CoVs	(97)
EGFR	EGFR kinase inhibitors (Erlotinib, Gefitinib and Osimertinib)	IAV	Erlotinib and Gefitinib reversibly bind to the ATP-binding pocket of EGFR, while Osimertinib covalently binds to Cys797 in the EGFR active site, inhibiting EGFR phosphorylation and thereby suppressing IAV internalization	(135)

## Glossary

25HC: 25-hydroxycholesterol
ACE2: angiotensin-converting enzyme 2
ACAT: acyl‐CoA: cholesterol acyltransferase
ADP: adenovirus death protein
AGRN: agrin
ALCAM: activated leukocyte cell adhesion molecule
AP2B1: adaptor protein 2 subunit beta 1
APN: aminopeptidase N
ARE: apical recycling endosome
AVP: adenovirus protease
CAR: coxsackievirus and adenovirus receptor
Cav-1: caveolin-1
CCPs: clathrin-coated pits
CH25H: cholesterol 25-hydroxylase
CIL: cytoplasmic intracellular loop
CME: clathrin-mediated endocytosis
CMK: decanoyl-RVKR-chloromethylketone
CoVs: coronaviruses
CTSL: cathepsin L
CtBP1: C-terminal binding protein 1
CX3CR1: C-X3-C motif chemokine receptor 1
DBP: DNA binding protein
DC-SIGN: dendritic cell-specific intercellular adhesion molecule-3-grabbing non-integrin and liver
DENV: dengue virus
DFMO: difluoromethylornithine
DPP4: dipeptidyl peptidase 4
DSG2: desmoglein-2
EEA1: early endosome antigen 1
EGFR: epidermal growth factor receptor
ESCRT: endosomal sorting complexes required for transport
FFAR2: free fatty acid receptor 2
GAGs: glycosaminoglycans
GE: gene end
GILT: gamma-interferon-inducible lysosomal thiol reductase
GPCR: G protein-coupled receptor
GPI: glycosylphosphatidylinositol
GS: gene start
HAdV: human adenovirus
HA: hemagglutinin
HIV: human immunodeficiency virus
HMPV: human metapneumovirus
HSPGs: heparan sulfate proteoglycans
HSV: herpes simplex virus
IAV: influenza A virus
IBs: inclusion bodies
IFITMs: interferon-inducible transmembrane proteins
IgSF: immunoglobulin superfamily
IL-8: Interleukin-8
KCa1.1: calcium-activated large-conductance potassium channel
L-SIGN: lymph node-specific ICAM-3 grabbing non-integrin
LY6E: lymphocyte antigen 6 family member E
mGluR2: metabotropic glutamate receptor 2
MLP: major late promoter
MLV: murine leukemia virus
NA: neuraminidase
NCOA7: Nuclear Receptor Coactivator 7
Neo1: neogenin
NLSs: nuclear localization signals
NPC: nuclear pore complex
ODC1: ornithine decarboxylase 1
PAK1: p21-activated kinase 1
PC: proprotein convertase
PSGL-1: P-selectin glycoprotein ligand-1
pTP: preterminal protein
RABGAP1L: RAB GTPase activating protein 1 like
Rac1: Ras-related C3 botulinum toxin substrate 1
RNP: ribonucleoprotein complex
RSV: respiratory syncytial virus
RTK: receptor tyrosine kinase
SCAP: severe community-acquired pneumonia
SERINC5: serine incorporator 5
SLC35B4: solute carrier family 35 member B4
SLC35: solute carrier family 35
TMPRSS2: transmembrane serine protease 2
UDP-GlcNAc: uridine diphosphate N-acetylglucosamine
UDP-xylose: uridine diphosphate xylose
UIMs: ubiquitin-interacting motifs
VCAP: viral community-acquired pneumonia
VLPs: virus-like particles
WNV: west Nile virus
ZIKV: Zika virus
